# Epidemiological and clinical profile of hepatitis B infection in ART-naïve people living with HIV in Maputo, Mozambique: a cross-sectional study

**DOI:** 10.1136/bmjph-2024-001563

**Published:** 2025-08-04

**Authors:** Lucia Mabalane Chambal, Charlotta Nilsson, Elias Manjate, Corssino Tchavana, Orvalho Augusto, Vanda dos Muchangos, Júlia Muando, Esperanca Sevene

**Affiliations:** 1 Faculty of Medicine, Eduardo Mondlane University, Maputo, Mozambique; 2 Maputo Central Hospital, Maputo, Mozambique; 3 Department of Laboratory Medicine, Division of Clinical Microbiology, Karolinska Institute, Stockholm, Stockholm, Sweden; 4 Public Health Agency of Sweden, Solna, Stockholm, Sweden; 5 Manhica Health Research Center, Manhica, Maputo, Mozambique; 6 Department of Global Health, University of Washington, Seattle, Washington, USA; 7 Department of Physiological Science, Clinical Pharmacology, Faculty of Medicine, Eduardo Mondlane University, Maputo, Maputo City, Mozambique; 8 National Institute of Health, Maputo, Mozambique

**Keywords:** HIV, Comorbidity, Epidemiology

## Abstract

**Introduction:**

Globally, more than 254 million people are living with hepatitis B virus (HBV), and 7.4% of the people living with HIV (PLHIV) are coinfected with HBV. More than 70% of them reside in Africa. We aimed to describe the characteristics of newly diagnosed antiretroviral treatment (ART)-naïve HIV/HBV co-infected and HIV monoinfected patients.

**Methods:**

This cross-sectional study included newly HIV-diagnosed ART-naïve patients recruited at Mavalane Health Centre located in a periurban area of Maputo City. Between May 2021 and November 2022, all patients over 18 years old were enrolled and screened for hepatitis B surface antigen (HBsAg). Data on socio-demographic and clinical characteristics, haematology, liver and kidney function tests, CD4+T cell counts, serological markers of hepatitis B (IgM Core hepatitis B antibody, hepatitis B e antigen, and hepatitis B e antibody), HIV and HBV viral loads were assessed using standard procedures.

**Results:**

A total of 1106 participants were included. The age of the participants ranged from 18 years to 71 years with a median of 34.0 (IQR: 28.0–42.0) years, 513 (46.4%) were men and HBsAg was reactive in 81 participants, yielding a co-infection rate of 7.3%. Being male (OR, 1.72; 95% CI, 1.06 to 2.83) or a sex worker (OR, 3.69; 95% CI, 1.10 to 10.58) was associated with the co-infection. The median Aspartate Aminotransferase-Platelet Ratio Index (APRI) of the HIV/HBV co-infected was 0.5 (IQR 0.3–1.1), with 40/81 (49.4%) presenting with an APRI>0.5. Overall, 67/81 (80.2%) of the co-infected people were HBeAg-negative. The median APRI was 0.5 (IQR, 0.3–1.1) for the HBeAg-negative and 0.7 (IQR 0.3–1.4) for the HBeAg-positive subjects. The median HBV-DNA was 258.0 IU (IQR, 10.0–4974.5) for the HBeAg-negative and 746 287.0 IU (IQR 2720.0–49 899 213.0) for the HBeAg-positive subjects. Two (3.0%) HBeAg-negative and one (7.1%) HBeAg-positive subjects presented with hepatocellular carcinoma.

**Conclusion:**

These data confirm the high prevalence of HIV/HBV co-infection in Mozambique and bring new data related to HBeAg status, reinforcing the need to test all PLHIV for HBV and to integrate the management and monitoring of hepatitis B and liver disease-specific tests in public ART programmes to predict and reduce the occurrence of HBV complications and mortality.

**WHAT IS ALREADY KNOWN IN THIS TOPIC**

**WHAT THIS STUDY ADDS**

- We found a high prevalence of HBV among ART-naïve people living with HIV (PLHIV) in southern Mozambique and possible associated risk factors.
- Furthermore, we document a high prevalence of HBeAg-negative subjects for the first time.

**WHAT THIS STUDY MIGHT AFFECT RESEARCH, PRACTICE OR POLICY**

- We recommend routine screening and HBV care management in PLHIV.

## Background

 Despite the worldwide availability of a safe and effective vaccine against hepatitis B virus (HBV) since 1981, and significant improvements in treatment, the global prevalence of chronic hepatitis B (CHB) infection remains high. CHB is a leading cause of cirrhosis, end-stage liver disease and hepatocellular carcinoma (HCC), leading to significant morbidity and mortality.^1^ In 2022, an estimated 254 million people worldwide were living with CHB, with 1.1 million deaths annually, primarily in low-middle-income countries (LMICs).^2^ Alarmingly, only 13% of the people living with HBV infection had been diagnosed, and only 3% were receiving treatment.^2^ About 5% of the WHO African Region (AFR) and Western Pacific Region populations are living with hepatitis B, yet only 18% of newborns received the hepatitis B birth-dose vaccination (HepB-BD).^2^ It is estimated that 7% of the people living with HIV (PLHIV) globally are co-infected with HBV, with over 70% residing in WHO AFR.^3^

The high prevalence of HBV infection in WHO AFR poses a significant burden related to vertical and perinatal transmission.^2 4^ In 2016, the World Health Assembly set a target of eliminating viral hepatitis as a public health problem by 2030, including the elimination of mother-to-child transmission (EMTCT) of HBV. The target of a 90% reduction in incidence, a 65% reduction in mortality from 2015 baseline levels and ≥90% coverage with both a timely HepB-BD and three doses of HepB (HepB3) was defined.^2 5 6^ Key strategies for accelerating progress toward this target include the introduction of HepB-BD, improving HepB3 coverage, enhanced access to diagnosis and effective monitoring of EMTCT implementation.^2 7^

Mozambique is endemic for both HBV and HIV infection. Studies conducted in different parts of the country have reported HBV infection rates of 9.1% and 7.6% among PLHIV initiating antiretroviral treatment (ART),^8 9^ 11.4% in prisoners living with HIV,^10^ 9.3% in blood donors,^11^ 32.8% in persons who use drugs,^12^ 12.2% in youths^13^ and 4.0% in pregnant women.^14^ Historically, Mozambique had one of the highest incidence rates of HCC worldwide, marked by an early-age presentation.^15^ A hospital-based study found that 56.2% (114/211) of diagnosed HCC had Hepatitis B, with HIV/HBV co-infection observed in 68% of cases, and PLHIV presented with HCC earlier than HIV-negative patients.^15 16^ The country implemented HepB3 in infants as part of its Extended Programme of Immunization (EPI) in 2001.^17^

In 2021, the national prevalence of HIV among adults in Mozambique was 12.5%.^18^ The country has adopted the WHO guidelines for treating all PLHIV. The first-line ART regimen includes tenofovir (TDF), which is also effective against HBV and is expected to reduce the mortality rates in PLHIV/HBV co-infection by lowering both viral loads.^19 20^ However, some studies have shown higher mortality rates in those with HIV/HBV co-infection compared with those with HIV or HBV monoinfection.^21^ Data on the serological and virological profiles of HBV in HIV/HBV co-infected patients are limited in Mozambique. This study aimed to describe the prevalence of HBV alongside sociodemographic and behavioural characteristics, as well as the clinical, laboratory, serological and virological profiles in newly HIV-diagnosed ART-naïve patients.

## Materials and methods

### Study design and setting

This was a cross-sectional study that included newly HIV-diagnosed ART-naïve patients, conducted at Mavalane Health Centre (MHC), located in a periurban area in Maputo City. Maputo City, the capital of Mozambique, is in the south of the country; by 2021, it had about 1 088 449 inhabitants and the third highest prevalence of HIV among adults in the country, 16.2%.^18^ MHC is a health centre that provides services to a population from a large health area in Mavalane. The health centre provides comprehensive care for HIV, including counselling, testing and treatment, following the ‘Treat All’ strategy within the Joint United Nations Programme on HIV/AIDS Goal target of 95-95-95. Data from the health centre records show that the centre has a median of 100 newly diagnosed HIV cases monthly and has cumulatively registered 8348 PLHIV on treatment since its opening in 2009 to June 2022. The recruitment into the study occurred between May 2021 and November 2022.

### Study population

All HIV-1 diagnosed ART-naïve subjects, at least 18 years old, who attended the clinic during the study period were invited to participate after receiving counselling and a diagnosis of HIV infection and consecutively enrolled after providing written informed consent. The study exclusion criteria were tuberculosis diagnosis, pregnancy, previously known liver disease and patients unwilling or unable to participate.

### Sample size consideration

Originally, the sample size was estimated with two considerations: (1) a previously reported prevalence of hepatitis B among PLHIV of 9.1%; (2) we wanted to detect a 50% (with a margin of error of 10%) viral suppression of hepatitis B by 12 months of follow-up. For a 5% error type-1, 80% power and 30% potential loss to follow-up, we needed 253 patients on treatment for hepatitis B among ART-naïve PLHIV. To find this sample, we needed to screen 2780 ART-naïve PLHIV. However, as the study was set up during the peak of the COVID-19 pandemic in Mozambique, some study health facilities were transformed into COVID-19 treatment centres. Additionally, there were restrictions on the circulation of people seeking health services, a shortage of individual protective equipment for health workers and significant barriers in acquiring reagents. Consequently, we were forced to limit the study to only one health facility and recruit all the potential participants consecutively for 18 months.

### Data collection and sample processing

Patients attending the MHC for clinical purposes or voluntary testing were first tested for HIV following the WHO testing protocol and the national algorithm for HIV testing, which consists of a sequential algorithm of two immunochromatographic assays. Determine HIV-1/2 (Alere, Japan) is used for screening, and UniGold HIV-1/2 (Trinity Biotech PLC, Ireland) is used to confirm seroreactivity.^22^ For all the eligible participants, demographic data, relevant information for assessing the possible risk factors for HIV and/or HBV infection and clinical data focused on liver disease infection were collected using a questionnaire administered by trained nurses after giving informed consent.

At the study site, fingerstick whole blood was used for Hepatitis B surface antigen (HBsAg) testing on the first visit, using the enzyme immunoassay-based rapid test Determine HBsAg 2 (Alere Medical, Chiba Japan (Now Abbott)).

Whole blood samples were collected aseptically by venipuncture to perform a haemogram with differentials using a Sysmex KX21N and biochemistry analyses (aspartate aminotransferase (AST), alanine aminotransferase (ALT), gamma-glutamyl transferase (GGT), total bilirubin, alkaline phosphatase (ALP), urea (Ur) and creatinine (Cr)) were performed using an automated chemistry analyser (Vitalab) at the emergency laboratory at Maputo Central Hospital. CD4^+^ T Cell analysis using a BD FacsCalibur analyser and HIV-1 and HBV viral load analysis using COBAS AMpliPrep/COBAS TaqMan 96 and COBAS 6800 analyser (Roche Diagnostics, USA) were performed at the National Institute of Health and hepatitis B serological assays (hepatitis B e antibody (anti-HBe), AgHBe and IgM Core hepatitis B antibody (anti-HBc IgM)) using a epatitis B profile kit RecombiLisa kit (CTK, California, USA) were performed at the Faculty of Medicine, Eduardo Mondlane University.

### Clinical assessments

Clinical data were collected through a summarised physical exam performed by trained nurses, which looked for body mass index and signs of liver disease, such as hepatomegaly, splenomegaly and ascites.

Kidney function was estimated using the race-free CKD-EPI equation, which estimates the glomerular filtration rate (eGFR) using Cr and age (26). We used a cut-off of 50 mL/min for analysis because people with impaired eGFR at baseline (<50 mL/min) should not initiate TDF treatment.^19^

For liver disease staging, we used two non-invasive tests (NIT), APRI (AST-Platelet Ratio Index) and FIB-4 (Fibrosis 4 Index), that are calculated using AST, ALT and platelet count: APRI (AST-Platelet Ratio Index); APRI = (AST/upper limit of normal) x 100)/ platelet count (10^9^ /L) and FIB-4: FIB-4 = (age (years) x AST (IU/L))/(platelet count (10^9^ /L x [ALT (IU/L)(1/2)]). According to 2024 WHO guidelines, the evidence of significant fibrosis should be based on an APRI score >0.5 and a FIB-4 score >3.25, and cirrhosis should be based on clinical criteria or an APRI score >1.0.^23^

### Definitions used as per WHO

- HBsAg: HBV envelope protein and excess coat particles detectable in the blood in acute and CHB infection.^23^
- Hepatitis B e antigen (HBeAg): viral protein found in the high replicative phase of hepatitis B. HBeAg is usually a marker of high replication levels with wild-type virus but is not essential for viral replication.
- Anti-HBe: antibody to HBeAg detected not only among people with lower levels of HBV replication but also in people with HBeAg-negative disease (HBV that does not express HBeAg).
- IgM anti-HBc: subclass of anti-HBc (antibody to hepatitis B core (capsid) protein). Detected by sensitive assays during acute hepatitis B and a flare or reactivation of chronic HBV.
- CHB: the presence of HBsAg in adults and the persistence of HBsAg in adolescents and children for 6 months or more.
- HBeAg-positive disease: characterised by the continued presence of HBeAg and relatively high serum HBV DNA concentrations (typically between 10^5^ IU/mL and 10^7^ IU/mL) but raised serum aminotransferases, indicating active inflammatory disease.
- HBeAg-negative disease: characterised by mutations in the precore or basal core promoter that downregulate HBeAg despite continued HBV replication. It is characterised by fluctuation in serum ALT. HBV DNA concentrations are lower than those found among HBeAg-positive people and typically between 10^3^ IU/mL and 10^5^ IU/mL but higher than those in HBeAg-negative infection.

### Variables

The primary outcome of this study was the hepatitis B coinfection (positivity to HBsAg). Also, the HBeAg positivity is an additional outcome. Further, in the case of positivity to HBV, we collected viral load data. For the study of factors associated with hepatitis B, the following variables were considered:

- Sociodemography: age at the date of recruitment (in years declared by the patient), gender (male or female), level of education (collected as either illiteracy, primary, secondary or higher education) and marital status (collected as single, married or divorced).
- Behavioural characteristics: unprotected sexual intercourse in the last 6 months (yes or no), the total number of partners in life (none, 1 or ≥2), being a sex worker (yes or no), having scarification (yes or no), having tattoos or piercings (yes or no), ever received blood transfusion (yes or no), whether consumed alcohol (yes or no) and the HIV status of the partner (positive, negative or does not know)
- Immunisation to hepatitis B: a question about previous vaccination to hepatitis B was asked (yes or no)

In addition, we collected physical exam variables and selected laboratory information to assess the clinical status of the patients:

- Haematological variables: haemoglobin (g/L), lymphocyte count per litre and platelets count per litre, CD4+T count (cells per mm^3^)
- Liver function: ALT in IU, AST in IU, GGT in IU and ALP in IU.
- Kidney function: Ur in µmol/L and Cr in µmol/L.

### Data management and analysis

Data from all study participants were collected using REDCap (2024 Vanderbilt University)^24 25^ and then exported to R V.4.3.1 for analysis. Descriptive statistics were used, with frequencies and percentages for categorical variables, means, SD and quantiles for quantitative variables. For the prevalence of HIV/HBV co-infection, exact 95% CIs are presented. We compare groups using Pearson’s χ^2^ or Fisher’s exact test for categorical variables, and the Wilcoxon rank sum test to compare medians. Furthermore, the association between each demographic variable and possible risk factors for infection and the prevalence of HIV/HBV co-infection was assessed as unadjusted and adjusted ORs estimated through logistic regression. For adjustment, potential risk factors (age, gender, level of education, marital status, unprotected sexual intercourse in the last 6 months, scarification, tattoo/piercings, alcohol consumption, received blood transfusions and number of sexual partners ever) are included in the multivariable model. The variance inflation factors (VIFs) were used to check for multicollinearity. No covariate had a VIF above 2. We report Wald-based values, 95% CIs and p values. As there was a small amount of missing data, we conducted a complete case analysis.

## Results

### Demographic and behavioural characteristics

**Figure 1:**
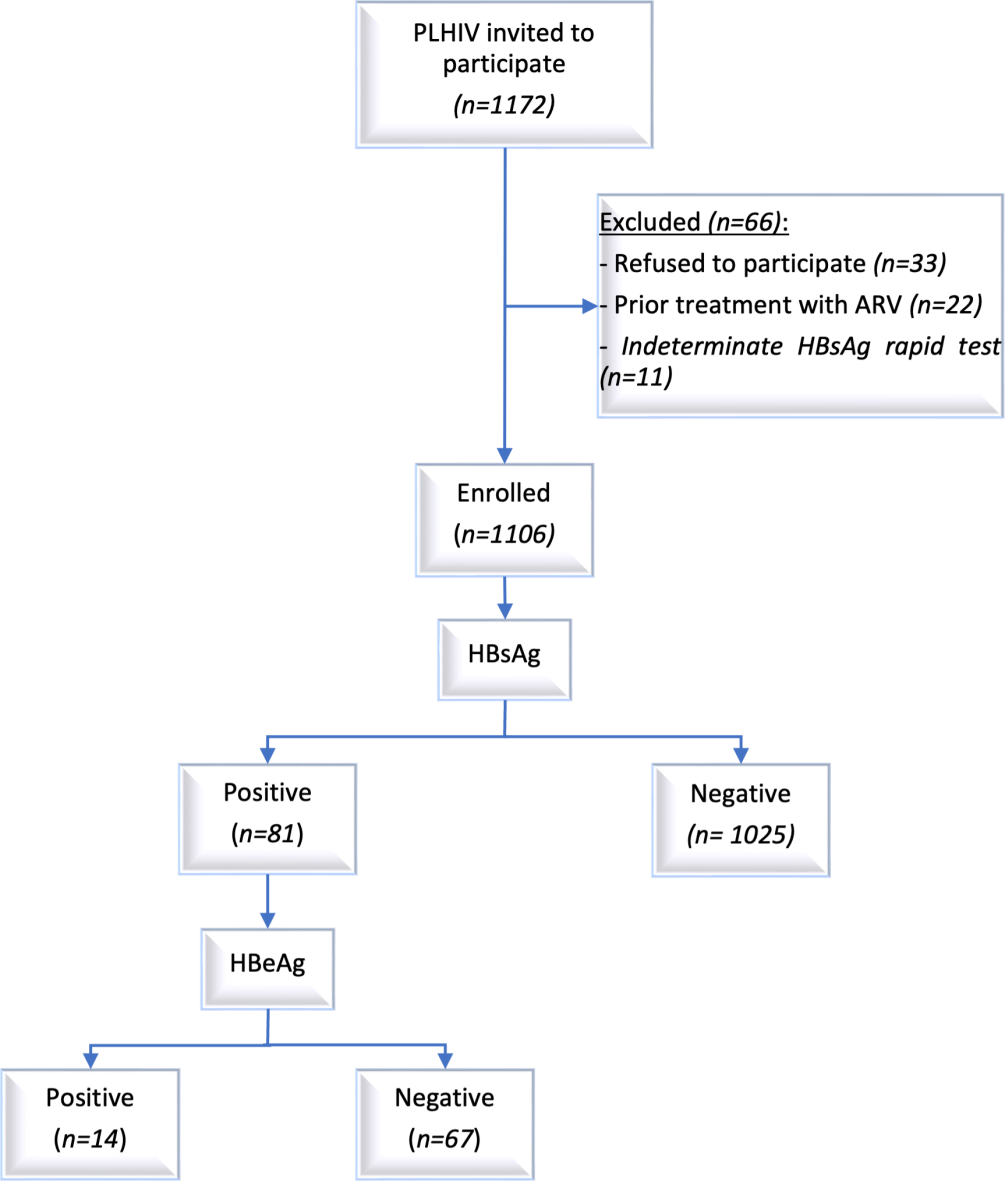
Flowchart of the participant’s recruitment and HBV screening. HBeAg, hepatitis B e antigen; HBsAg, hepatitis B surface antigen; PLHIV, people living with HIV.

A total of 1106 ART-naïve HIV-infected participants were enrolled in the study (figure 1). The age of the participants ranged from 18 years to 71 years with a median of 34.0 (IQR: 28.0–42.0) years, 53.6% were women, 53.7% were married and 52.4% had secondary or higher education. Table 1 shows the demographic characteristics of the participants. Online supplemental tables 1-3 show the demographic characteristics per gender of the overall participants (S1), among HIV+/HBV+participants (S2) and among individuals aged 18–24 years categorised by whether they were born before or after 2001, the year when HepB3 vaccination began in Mozambique (S3).

**Table 1:** Sociodemographic characteristics of study subjects and prevalence of HIV/HBV co-infection

	Overall N (%)	HIV and HIV/HBV co-infection		Prevalence of co-infection (%)*	95% CI
		HIV+/HBV- N (%)	HIV+/HBV+ N (%)		
Total	1106 (100.0)	1025 (93.0)	81 (7.0)	7.3	5.9 to 9.0
Age (years)					
Median (IQR)	34.0 (28.0, 42.0)	35.0 (28.0, 42.0)	34.0 (30.0, 39.0)		
Age (years), n (%)					
18–24	146 (13.2)	139 (13.6)	7 (8.6)	4.8	2.0 to 9.6
25–34	410 (37.1)	371 (36.2)	39 (48.2)	9.5	6.9 to 12.8
35–44	353 (31.9)	328 (32.0)	25 (30.7)	7.1	4.6 to 10.3
>=45	197 (17.8)	187 (18.2)	10 (12.4)	5.1	2.5 to 9.1
Gender, n (%)					
Male	513 (46.4)	465 (45.4)	48 (59.3)	9.4	7.0 to 12.2
Female	593 (53.6)	560 (54.6)	33 (40.7)	5.6	4.0 to 8.0
Level of education, n (%)					
Primary/illiteracy	526 (47.6)	487 (47.5)	39 (48.2)	7.4	5.3 to 10.0
Secondary/higher	580 (52.4)	538 (52.5)	42 (51.9)	7.2	5.3 to 10.0
Marital Status, n (%)					
Married	594 (53.7)	548 (53.5)	46 (56.8)	7.7	6.0 to 10.0
Single	480 (43.4)	447 (43.6)	33 (40.7)	6.9	5.0 to 10.0
Divorced	32 (2.9)	30 (2.9)	2 (2.8)	6.3	0.8 to 21.0
Unprotected sexual intercourse in the last 6 months**,** n (%)					
Yes	525 (47.5)	485 (47.3)	40 (49.4)	7.6	6.0 to 10.2
No	581 (52.5)	540 (52.7)	41 (50.6)	7.1	5.1 to 9.5
Sex worker, n (%)					
Yes	21 (1.9)	16 (1.7)	5 (6.2)	23.8	8.2 to 47.2
No	1085 (98.1)	1009 (98.4)	76 (93.8)	7.0	5.6 to 8.7
Scarification, n (%)					
Yes	38 (3.4)	34 (3.3)	4 (4.9)	10.5	3.0 to 24.8
No	1068 (96.6)	991 (96.7)	77 (95.1)	7.2	5.7 to 8.93
Tattoo/piercings, n (%)					
Yes	193 (17.5)	174 (17.0)	19 (23.5)	9.8	6.0 to 15.0
No	913 (82.6)	851 (83.0)	62 (76.5)	6.8	5.3 to 8.6
Received blood transfusion, n (%)					
Yes	33 (3.0)	28 (2.73)	5 (6.17)	15.2	5.1 to 31.9
No	1073 (97.0)	997 (97.27)	76 (93.83)	7.1	5.6 to 8.8
Prior HBV vaccination, n (%)					
Yes	56 (5.1)	53 (5.17)	3 (3.7)	5.4	1.1 to 14.9
No	1050 (95.0)	972 (94.83)	78 (96.3)	7.4	5.9 to 9.2
Alcohol consumption, n (%)					
Yes	556 (50.3)	509 (49.7)	47 (58.0)	8.5	6.3 to 11.1
No	550 (49.8)	516 (50.3)	34 (42.0)	6.2	4.3 to 8.5
Number of partners, n (%)					
None	202 (18.3)	188 (18.3)	14 (18.2)	6.9	3.8 to 11.4
1	883 (79.8)	820 (80.0)	63 (81.8)	7.1	5.5 to 9.0
≥2	21 (1.9)	17 (1.7)	4 (4.9)	19.0	5.4 to 41.9
HIV partner status, n (%)†					
Positive	240 (25.6)	219 (26.2)	21 (31.3)	8.8	5.5 to 13.1
Negative	116 (12.8)	110 (13.1)	6 (9.0)	5.2	1.9 to 10.9
Unknown	548 (60.6)	508 (60.7)	40 (59.7)	7.3	5.3 to 9.8

* Prevalence of coinfection is the proportion of HBsAg positive among HIV patients.

† Only participants who are married or have a partner are included.

HBsAg, hepatitis B surface antigen; HBV, hepatitis B virus.

Regarding the risk factors for HIV and/or HBV infection (table 1), 47.5% of the participants reported unprotected sexual intercourse with an occasional partner within 6 months before recruitment, 1.9% reported being a sex worker (66.7% women vs 23.7% men) and 1.9% reported having more than one sexual partner. Half of the participants (50.3%) reported use of alcohol, 17.5% reported having a tattoo/piercing and 3.0% reported having received a blood transfusion. Among participants with a partner, 60.6% did not know the HIV status of the partner. Of the participants, 5.1% reported prior HBV vaccination. None of the participants reported a prior history of hepatitis B testing or diagnosis.

### Hepatitis B co-infection prevalence and risk factors

The overall prevalence of hepatitis B co-infection was 7.3% (81/1106; 95% CI, 5.9 to 9.0%), whereas the prevalence in men and women was 9.4% (48/513; 95% CI, 7.0 to 12.2%) and 5.6% (33/593; 95% CI, 4.0 to 8.0%), respectively. By age, an inverted-U shape is observed with the under 25 years and above 45 years showing a prevalence of 4.8% (7/146; 95% CI, 2.0 to 9.6%) and 5.1% (10/197; 95% CI, 2.5 to 9.1%), respectively; and the age categories 25–34 years and 35–44 years showing 9.5% (39/410; 95% CI: 6.9 to 12.8%) and 7.1% (25/353; 95% CI: 4.6 to 10.3%), respectively (table 1 and online supplemental figure 1). Online supplemental table 4 shows the prevalence of hepatitis B among PLHIV aged 18–24 years at the time of recruitment.

Table 2 shows the association between demographic characteristics and the prevalence of HBV infection. Gender and being a sex worker were independently associated with co-infection and, after adjustment, virtually remained at the same strength and direction. Compared with women, men had 72% higher odds (OR, 1.72; 95% CI, 1.06 to 2.83, p=0.030) of being co-infected, whereas sex workers had more than three times higher odds (OR, 3.69; 95% CI, 1.10 to 10.58, p=0.021) of being co-infected compared with non-sex workers. There was no association between unprotected sex in the last 6 months and HBV infection (OR, 0.94; 95% CI, 0.27 to 2.98, p=0.971). The direction and strength of the associations did not materially change when prevalence ratios were used instead of ORs (online supplemental table 5) despite many numerical fitting issues.

**Table 2:** Sociodemographic characteristics of study subjects and risk factors for HIV/HBV co-infection

Characteristic	Unadjusted			Adjusted		
	OR	95% CI	P value	OR	95% CI	P value
Age (years)			0.132			0.234
18–24	0.48	0.19 to 1.03	0.081	0.53	0.18 to 1.31	0.204
25–34	1.00	—	—	1.00	—	—
35–44	0.73	0.42 to 1.22	0.229	0.70	0.41 to 1.20	0.201
≥45	0.51	0.24 to 1.00	0.065	0.52	0.23 to 1.09	0.098
Gender						
Male	1.00	—		1.00	—	—
Female	1.75	1.11 to 2.80	0.017	1.72	1.06 to 2.83	0.030
Level of education						
Primary/illiteracy	1.00	—		1.00	—	
Secondary/higher	0.97	0.62 to 1.54	0.912	0.94	0.59 to 1.51	0.794
Sex worker						
No	1.00	—			—	—
Yes	4.15	1.33 to 10.9	0.007	3.69	1.10 to 10.58	0.021
Blood transfusion						
No	1.00	—		1.00	—	—
Yes	2.34	0.78 to 5.76	0.089	1.79	0.53 to 4.99	0.297
Prior HBV vaccination						
No	—	—		—	—	—
Yes	0.71	0.17 to 1.97	0.564	1.16	0.24 to 4.32	0.837
Marital status			0.839			0.899
Single	1.00	—		1.00	—	—
Married	1.14	0.72 to 1.82	0.588	1.09	0.67 to 1.81	0.728
Divorced	0.90	0.14 to 3.18	0.892	1.34	0.20 to 5.27	0.716
Number of partners			0.140			0.259
None	1.00	—		1.00	—	
1	0.60	0.23 to 1.39	0.919	0.90		0.738
≥2	0.76	0.45 to 1.31	0.064	2.35		0.197
HIV partner status			0.476			
Positive	1.76	0.69 to 4.48	0.289			
Negative	1.00	—	0.261			
Unknown	1.44	0.60 to 3.49	0.546			
Unprotected sexual intercourse in the last 6 months						
No	1.00	—		1.00	—	
Yes	1.09	0.69 to 1.71	0.720	0.94	0.27 to 2.98	0.971
Scarification						
No	1.00	—		1.00	—	
Yes	1.51	0.44 to 3.92	0.444	1.02	0.27 to 2.98	0.971
Tattoo/piercings						
No	1.00	—		1.00	—	
Yes	1.50	0.85 to 2.52	0.141	1.21	0.65 to 2.17	0.528
Alcohol consumption						
No	1.00	—		1.00	—	
Yes	1.40	0.89 to 2.23	0.149	1.14	0.70 to 1.88	0.606

HBV, hepatitis B virus.

### Clinical and laboratory characteristics

Regarding the clinical signs of liver disease described in online supplemental table 6, 4/10 (40%) of the participants who presented with jaundice, 3/4 (75%) of the participants who presented with ascites and 8/11 (72.7%) of the participants who presented with hepatomegaly were co-infected.

**Figure 2:**
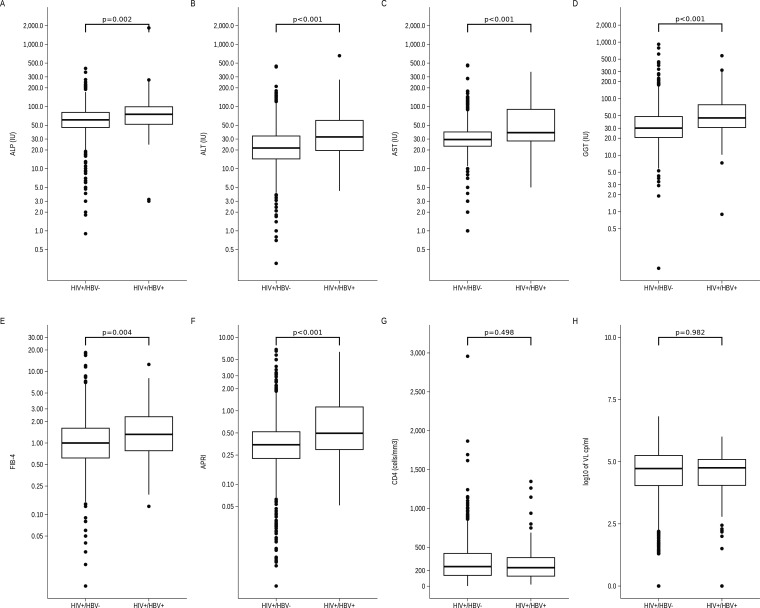
Comparison of laboratory parameters between HIV+/HBV- (mono-infected) and HIV+/HBV+ (co-infected): (A) for alkaline phosphatase (ALP); **(B**) for alanine transferase (ALT); (**C**) for aspartate aminotransferase (AST); **(D**) for gamma-glutamyl transferase (GGT); **(E**) for FIB-4 (Fibrosis-4 Score); **(F**) for APRI (AST-Platelet Ratio Index); **(G**) for CD4 cell counts and (H) for HIV viral load in log10 scale. HBV, hepatitis B virus.

The laboratory characteristics of HIV-monoinfected and HIV/HBV co-infected individuals are presented in online supplemental table 7 and figure 2. The median AST was 29.5 IU (IQR 23.0–39.0) for the HIV monoinfected participants and 38.0 IU (IQR 27.9–90.0) for the co-infected participants (p<0.001). The median ALT was 21.5 IU (IQR 14.4–33.6) for the HIV monoinfected and 32.4 IU (IQR 19.6–59.8) for the co-infected participants (p<0.001). The median APRI was 0.3 (IQR 0.2–0.5) for the HIV monoinfected and 0.5 (IQR 0.3–1.1) for the co-infected participants (p<0.001), with 210 (26.5%) and 40 (49.5%) presenting with an APRI>0.5 for the HIV monoinfected and co-infected participants, respectively (p<0.001). The median FIB-4 was 1.00 (IQR 0.6–1.6) for the HIV monoinfected, and 1.3 (IQR 0.8–2.3) for the co-infected participants (p=0.004), with 73 (7.2%) and 12 (14.8%) with a FIB-4 >3.25 for the HIV monoinfected and co-infected participants (p=0.013). Regarding kidney function, 67 (6.5%) monoinfected and 3 (3.7%) co-infected participants had an eGFR <50 mL/min/173 m^2^ (p=0.475).

Using 2015 WHO guidelines, currently in use in Mozambique, 8.6% (7/81) of participants were eligible for HBV treatment based on APRI>2.0, while 29.6% (24/81) were eligible based on HBV-DNA levels>20 000 IU/mL.^26 27^ Lowering treatment thresholds as now indicated in the 2024 WHO guidelines, APRI>0.5 and HBV-DNA >2000 IU/mL, increased eligibility to 49.4% (40/81) and 38.3% (31/81), respectively.^23^

The median CD4+T cell count was 252.0 cells/mm^3^ (IQR: 140.0–421.0) for the HIV monoinfected, and it was not substantially different from the median 238.0 cells/mm^3^ (IQR: 129.0–367.0) among the co-infected participants (p=0.486). Of the participants, 176 (17.2%) HIV monoinfected and 18 (22.2%) co-infected had a CD4+T cell count <100 cells/mm^3^ (p=0.615). The median HIV-RNA levels in the log scale was 4.7 (IQR: 4.0–5.2) for the HIV mono-infected and 4.8 (IQR: 4.0–5.1) for the co-infected participants (p=0.982). Also, 43 (4.3%) HIV monoinfected and four (4.9%) co-infected participants had undetectable HIV-RNA levels (p=0.595).

### Clinical and laboratory characteristics of the HIV/HBV co-infected patients by HBeAg status

To characterise the HBV replicative status, we screened the HIV/HBV co-infected patients (n=81) for HBeAg. Overall, 67 (80.2%) were HBeAg-negative, and 14 (19.8%) were HBeAg-positive, as presented in table 3. Among the HBeAg-negative patients, 36 (53.7%) were men, and among the HBeAg-positive patients, 12 (85.7%) were men (p=0.027).

**Table 3:** Demographic and laboratory characteristics of HBeAg-positive and HBeAg-negative participants

	HIV+/HBV+ N (%)	HBeAg-negative N (%)	HBeAg-positive N (%)	P value
Total	81 (100)	67 (80.2)	14 (19.8)	
Age				0.361*
Median	34.0 (30.0, 39.0)	34.0 (29.5, 40.0)	32.5 (30.3, 37.5)	
Gender				0.027†
Female	33 (40.7%)	31 (46.3%)	2 (14.3%)	
Male	48 (59.3%)	36 (53.7%)	12 (85.7%)	
CD4+T cell count, cells/mm^3^				0.745*
Median (IQR)	238.0 (129.0, 367.0)	232.0 (134.0, 367.5)	252.0 (94.5, 338.3)	
CD4+T**,** n (%)				0.695†
> 500	12 (14.8%)	9 (13.4%)	3 (21.4%)	
200–500	36 (44.4%)	31 (46.3%)	5 (35.7%)	
100–200	15 (18.5%)	13 (19.4%)	2 (14.3%)	
< 100	18 (22.2%)	14 (20.9%)	4 (28.6%)	
AST, IU, median (IQR**)**	38.0 (27.9, 90.0)	38.0 (28.4, 78.0)	41.3 (22.8, 112.8)	0.930*
AST, IU, n (%)				0.344†
≤30	25 (30.9%)	19 (28.4%)	6 (42.9%)	
>30	56 (69.1%)	48 (71.6%)	8 (57.1%)	
ALT, IU, median (IQR**)**	32.4 (19.6, 59.8)	30.4 (18.8, 52.4)	47.9 (20.4, 74.6)	0.359*
ALT, IU, n (%)				0.356‡
≤30	38 (46.9%)	33 (49.3%)	5 (35.7%)	
>30	43 (53.1%)	34 (50.7%)	9 (64.3%)	
GGT, IU, median (IQR**)**	45.6 (30.9, 78.0)	44.3 (30.8, 83.2)	51.3 (35.1, 73.7)	0.604*
GGT, IU, n (%)				0.573‡
≤49	46 (56.8%)	39 (58.2%)	7 (50.0%)	
>49	35 (43.2%)	28 (41.8%)	7 (50.0%)	
ALP, IU, median (IQR**)**	75.0 (51.8, 99.0)	71.5 (51.1, 99.5)	83.5 (77.0, 88.6)	0.465*
(Missing)	8	4	4	
ALP, IU, n (%)				1.000†
≤120	60 (82.2%)	52 (82.5%)	8 (80.0%)	
>120	13 (17.8%)	11 (17.5%)	2 (20.0%)	
(Missing)	8	4	4	
HIV-RNA (cp/mL) (log10)				0.212*
Median (IQR)	4.8 (4.0, 5.1)	4.7 (4.1, 5.0)	5.0 (4.1, 5.6)	
HIV-RNA (cp/mL), n (%)				0.844†
Undetectable	4 (4.9%)	3 (4.5%)	1 (7.1%)	
<300	6 (7.4%)	5 (7.5%)	1 (7.1%)	
300–1000	1 (1.2%)	1 (1.5%)	0 (0.0%)	
>1000	70 (86.4%)	58 (86.6%)	12 (85.7%)	
APRI				0.676*
Median (IQR)	0.5 (0.3, 1.1)	0.5 (0.3, 1.1)	0.7 (0.3, 1.4)	
APRI, n (%)				0.523‡
0.5	41 (50.6%)	35 (52.2%)	6 (42.9%)	
0.5–1.0	16 (19.8%)	32 (47.8%)	8 (57.1%)	
>1.0	24 (29.6%)			
FIB-4				0.990*
Median (IQR)	1.3 (0.8, 2.3)	1.2 (0.8, 2.6)	1.5 (0.8, 2.2)	
FIB-4, n (%)				1.000†
≤3.25	69 (85.2%)	57 (85.1%)	12 (85.7%)	
>3.25	12 (14.8%)	10 (14.9%)	2 (14.3%)	
Anti-HBc IgM, n (%)				0.439†
Negative	78 (96.3%)	65 (97.0%)	13 (92.9%)	
Positive	3 (3.7%)	2 (3.0%)	1 (7.1%)	
Anti-HBe, n (%)				<0.001‡
Negative	31 (38.3%)	17 (25.4%)	14 (100.0%)	
Positive	50 (61.7%)	50 (74.6%)	0 (0.0%)	
HBV DNA, IU/mL				<0.001*
Median (IQR)	813.0 (10.0, 32,700.0)	258.0 (10.0, 4,974.5)	746 287.0 (2,720.0, 49,899,213.0)	
HBV DNA, IU/mL, n (%)				0.002†
Undetectable	23 (28.4%)	23 (34.3%)	0 (0.0%)	
≤10	3 (3.7%)	3 (4.5%)	0 (0.0%)	
10–2000	24 (29.6%)	21 (31.3%)	3 (21.4%)	
2000–20 000	7 (8.6%)	6 (9.0%)	1 (7.1%)	
≥20 000	24 (29.6%)	14 (20.9%)	10 (71.4%)	

* Wilcoxon rank sum test.

† Fisher’s exact test.

‡ Pearson’s χ2 test.

ALP, alkaline phosphatase; ALT, alanine transferase ; Anti-HBe, Hepatitis B e antibody; APRI, AST-Platelet Ratio Index; AST, aspartate aminotransferase; FIB-4, Fibrosis-4 score; GGT, gamma-glutamyl transferase; HBeAg, Hepatitis B e antigen; HBV, hepatitis B virus ; IgM anti-HBc, gM Core hepatitis B antibody.

Although the median CD4+T cell count, median HIV-RNA (in log scale), median AST, median ALT, median GGT, median ALP and median FIB-4 were higher in HBeAg-positive patients compared with HBeAg-negative patients, these differences were not statistically significant. The median APRI was 0.5 (IQR 0.3–1.1) for the HBeAg-negative subjects with 32 (47.8%) presenting with an APRI>0.5 and 0.7 (IQR 0.3–1.4) for the HBeAg-positive subjects with 8 (57.1%) presenting with an APRI>0.5 (p=0.676 and p=0.523).

The median HBV-DNA viral load was 258.0 IU (IQR 10.0–4974.5) for the HBeAg-negative and 746 287.0 IU (IQR 2720.0–49,899,213.0) for the HBeAg-positive subjects (p=0.001). Among the HBeAg-negative, 44 (65.7%) had a detectable HBV-DNA, with 20 (29.9%) presenting with HBV-DNA >2000 IU and 14 (20.9%) with an HBV-DNA >20 000 IU and all the HBeAg-positive patients had an HBV-DNA >2000 IU (p=0.002). Three co-infected participants were identified with signs of HCC; two (3.0%) were HBeAg-negative, and one (7.1%) was positive.

## Discussion

The high prevalence of hepatitis B remains a significant public concern in WHO Africa and Pacific Asia Regions and is a general burden concerning vertical and perinatal transmission. Despite implementing HepB3 vaccination in Mozambique in 2001, this study revealed a high prevalence of HBV infection among the PLHIV. Male gender and sex work were identified as critical risk factors for HBV infection in this population. A large proportion of participants reported not being vaccinated. This study also provides the first report on the clinical and laboratory profiles associated with different replicative states, highlighting a notable prevalence of HBeAg-negative patients.

The prevalence of HBV in the present study (7.3%) was similar to the prevalence of HBV among the general population in the WHO AFR, which is 7.5%.^2^ The findings are consistent with data reported in Mozambique, where previous data reported HBV infection rates of 9.1% and 7.6% in PLHIV ART-naïve in the north and southern regions of the country, respectively, and confirms that Mozambique remains a country endemic for HBV.^8 9^ The prevalence of HBV was similar in all age groups older than 25 years. These data are consistent with previous studies in Mozambique and South Africa and suggest that our study subjects did not benefit from routine immunisation against HBV that started in Mozambique in 2001.^38^,^10 28^ Despite having established specific guidelines for the prevention, diagnosis and treatment of chronic hepatitis in 2019 and adopted the ‘WHO Triple Elimination Initiative’ targeting EMTCT of HIV, syphilis and hepatitis B, Mozambique has not yet implemented strategies such as routine HBV screening of pregnant women, high-risk population screening and access to treatment for people living with CHB.^23^ The lack of funding for viral hepatitis programmes has restricted implementing these strategies.

In this study, 94.8% of monoinfected and 96.3% of co-infected subjects reported not receiving HBV vaccination. Additionally, we found that 8.6% of the co-infected subjects were between 18–24 years old, and only half of these patients reported receiving an HBV vaccination. Mozambique is highly endemic for hepatitis B but has not implemented the HepB-BD and has a low coverage of HepB3 at 61% in 2021. In 2022, in East and South WHO AFR, only Botswana, Namibia, Mauritius and Uganda incorporated the HepB-BD into their EPI.^7^ Since 2009, WHO has recommended that all infants receive 3–4 doses of HBV vaccine, with the first dose administered as soon as possible after birth, preferably within 24 hours.^23^ Given the high prevalence of HIV/HBV co-infection and the low vaccination rates observed in this study, we strongly recommend the country to follow the WHO recommendations by increasing the coverage of HBV immunisation and incorporating HepBD into the EPI.

Although we had more women than men who were living with HIV in our cohort (53.6% vs 46.4%), the HBV prevalence was higher among men compared with women (9.4% vs 5.6%), consistent with findings from other studies from settings where perinatal transmission is dominant, such as in Taiwan and China,^29 30^ as well as in HIV cohort studies conducted in South Africa and Nigeria.^28 31^ The higher rates in men are likely due to behavioural risks, an increased likelihood of persistent HBV infection leading to chronicity, the effects of androgen hormones and the fact that women typically develop more intense innate, humoral and cellular immune responses to viral infections and vaccinations compared with men.^12 32 33^

Being a sex worker was also associated with co-infection, with a prevalence of 23.8%, which is higher than that described in a systematic review and meta-analysis of the global prevalence of HIV, HCV and HBV which showed a prevalence of co-infection of 7% in female sex workers in WHO AFR.^34^ WHO recommends that in settings with ≥2% or ≥5% a HBsAg seroprevalence in the general population, all adults should have routine access to and be offered HBsAg serological testing, especially those at high risk, such as people who inject drugs, people in prisons and other closed settings, men who have sex with men, sex workers, PLHIV and partners and pregnant women.^23^ Given the high prevalence of HIV/HBV co-infection in this study, particularly among men and sex workers, we strongly recommend the routine screening of high-risk individuals, such as men, PLHIV and sex workers, as well as HBV vaccination for non-immune high-risk population.^23^ These strategies should use existing facility-based (such as antenatal clinics and HIV or TB services) or community-based services for testing and vaccinating opportunities.

In our study, the median CD4+T cell of the co-infected patients was lower than that of those with monoinfection, although the difference was not statistically significant (238 cells/mm^3^ vs 252 cells/mm^3^, p=0.486). These results were consistent with previous studies conducted in Mozambique, Zambia and India involving ART-naïve PLHIV, but may be limited by an unknown duration of HIV disease.^8 9 35^ The impact of HIV/HBV co-infection on HIV disease progression remains controversial. While some studies found that HBV infection does not influence HIV progression,^36^ the majority suggests a strong association between HBV infection and HIV progression, with HIV/HBV co-infection affecting the course or control of HIV disease, being associated with advanced HIV disease and increased mortality, particularly in ART-naïve PLHIV.^3537^,^40^ It is suggested that a persistent state of immune activation in patients with chronic HBV infection can upregulate HIV replication.^39^ We strongly recommend hepatitis B screening in PLHIV as a strategy to prevent the mortality associated with HIV/HBV co-infection.

In our study, co-infected patients had a significant increase in AST, ALT, GGT and ALP compared with HIV monoinfected (p<0.001), which is consistent with data available from other studies done in Mozambique, Zambia, South Africa and Ethiopia.^8 9 41 42^ Evidence suggests that HIV infection can impact HBV infection with a rapid progression of the liver-related disease, higher serum HBV-DNA, lower rates of loss of HBeAg, increased risk of cirrhosis, HCC, liver-related mortality and an increased risk of hepatotoxicity to antiretroviral drugs especially at lower CD4+T cell counts.^37 38^ Elevated serum transaminases, especially ALT, strongly predict liver inflammation and are a risk factor for progressive liver disease and poor outcomes.^43 44^ The probability of evolution to cirrhosis increases in patients with moderate to severe inflammatory changes.

In this study, we also assessed the prevalence of liver fibrosis using NITs such as APRI and FIB-4 scores, which ensure timely detection and follow-up of liver complications.^45 46^ Studies done in the context of LMICs showed that APRI and FIB-4 are accurate, simple, cheap and readily available methods for assessing liver fibrosis in patients with HBV infection.^47^ The prevalence of liver fibrosis was 49.4% and 14.8% in the co-infected patients when using an APRI score >0.5 and a FIB-4 >3.25, respectively, which was higher than the HIV monoinfected, 26.5% and 7.2% using the same cut-offs (p*=*0.001 and p=0.013, respectively), confirming findings from other studies done in WHO AFR.^48 49^

The WHO recommends rapid initiation of ART with TDF-containing regimens in PLHIV and CHB, along with annual monitoring using NITs such as APRI score or transient elastography to assess the stage of disease and progression of fibrosis or cirrhosis. Monitoring ALT, AST and HBV DNA levels (when available) and HBeAg/anti-HBe is also advised.^23^ Based on our results, we recommend the country prioritise specialised delivery services for HIV/HBV co-infected patients to reduce the high mortality associated with this co-infection.

Liver fibrosis was also observed in monoinfected patients, and it has also been documented in studies done in Tanzania and Uganda which found a high prevalence of liver fibrosis in HIV monoinfected patients without viral hepatitis or tuberculosis. This highlights the need for further aetiological research on liver disease in African settings, considering tropical infections (eg, schistosomiasis, parasitic eosinophil-mediated liver disease) and environmental exposures such as alcohol and aflatoxins.^48 50^

In our study, we observed a higher prevalence of HBeAg-negative patients than HBeAg-positive patients (80.2% vs 19.8%), which aligns with findings from other regional studies. This characteristic is associated with HBV Genotype A1, the most prevalent genotype in Mozambique and this region.^39 51 52^ HBeAg-positive co-infected patients exhibited higher median HIV-RNA, HBV-DNA, AST, ALT and APRI compared with HBeAg-negative patients, as well as a greater proportion of individuals with CD4+T cell counts <200 cells/mm^3^, and these results were similar to previous studies conducted in the region.^51 52^

These findings suggest a potential for rapid progression of both HIV and HBV diseases. Previous studies indicate that HBeAg-negative individuals, despite having lower HBV-DNA, can experience a variable disease course characterised by fluctuating ALT and HBV-DNA levels, higher intrahepatic necroinflammatory lesions and accelerated progression to cirrhosis (annual rate 8–20%) and/or HCC, indicating a more robust immune response in HBeAg-negative infection.^23 27 51 53^ Although our study numbers are very low, we identified two HBeAg-negative and one HBeAg-positive patients with clinical signs of HCC. These findings are consistent with data from Egyptian and Greek cohorts and underscore the need for close monitoring of HBV infection, particularly in PLHIV.^51 54^

Making data accessible is one of the strategic directions toward the goal of eliminating hepatitis B as a public health problem by 2030. The data from this study can enhance the existing knowledge and strengthen country data systems, thereby improving accountability for HBV local and regional management. Conducting longitudinal studies to track the progression of HIV/HBV co-infection over time will provide valuable insights into how these infections interact, the effectiveness of treatment and long-term clinical outcomes.

This study has some limitations. It was initially planned to be conducted in three health centres; however, due to the COVID-19 pandemic, two of these centres were designated for COVID-19 management and care. The remaining health centre serves a high volume of patients within a periurban area, which could undermine comparability to other parts of Mozambique. However, being in a periurban area in the country’s capital city allows some comparability to national large villages and municipalities. Due to COVID-19-associated movement restrictions, we had low recruitment rates during 2021 and the beginning of 2022 and limited access to laboratory reagents, which led to some missing laboratory data observed. We did not collect an exhaustive list of risk factors to reduce interference with healthcare providers, and there is also a lack of standardised tools to collect this information for the Mozambican context. Finally, the vaccination history was self-reported by participants rather than based on a documented proof of vaccination records. This self-reported nature may introduce a potential recall bias, as individuals may not accurately remember their vaccination status or misreport their vaccination history, which may have limited our ability to assess the role of vaccination. In addition, we could not find subtle clinical signs of chronic liver disease.

## Conclusions

This study highlights the high prevalence of HBV in Mozambique, confirming HBV’s endemic status in the region. Despite the implementation of HepB3 in 2001, low vaccination rates and a significant number of unvaccinated individuals reveal critical gaps in public health efforts. Male gender and sex work emerged as significant risk factors for HBV infection, and co-infection was associated with severe liver disease. The high prevalence of HBeAg-negative disease suggests significant clinical implications, including higher HIV-RNA, HBV-DNA levels and liver enzymes, indicating accelerated disease progression. The observed liver fibrosis among co-infected patients poses a need to monitor these patients for timely intervention to reduce morbidity and mortality caused by HIV/HBV co-infection. In response to these findings, we strongly recommend that Mozambique prioritise HBV screening and vaccination, including introduction of the HepB-BD, into the EPI, using existing community or health-facility-based opportunities or programmes such as antenatal, HIV and TB clinics.

To attain the 2030 goals, it is imperative to reduce the global costs of HepB-BD, diagnostic tests and treatment. Additionally, it is crucial to diminish the stigma associated with HIV and HBV, thereby encouraging individuals to seek diagnosis and treatment, making it accessible in primary care settings or community-based clinics.

## Supplementary material

10.1136/bmjph-2024-001563 online supplemental file 1

## Data Availability

Data are available in a public, open access repository. All data relevant to the study are included in the article or uploaded as supplementary information.
