# Supplementary material for: Epidemiological and clinical profile of hepatitis B infection in ART-naïve people living with HIV in Maputo, Mozambique: a cross-sectional study
Source: BMJ Public Health. 2025 Aug 4;3(2):e001563. doi: 10.1136/bmjph-2024-001563 (PMC12323539; doi:10.1136/bmjph-2024-001563)
Supplement: online supplemental file 1 [file bmjph-3-2-s001.docx]

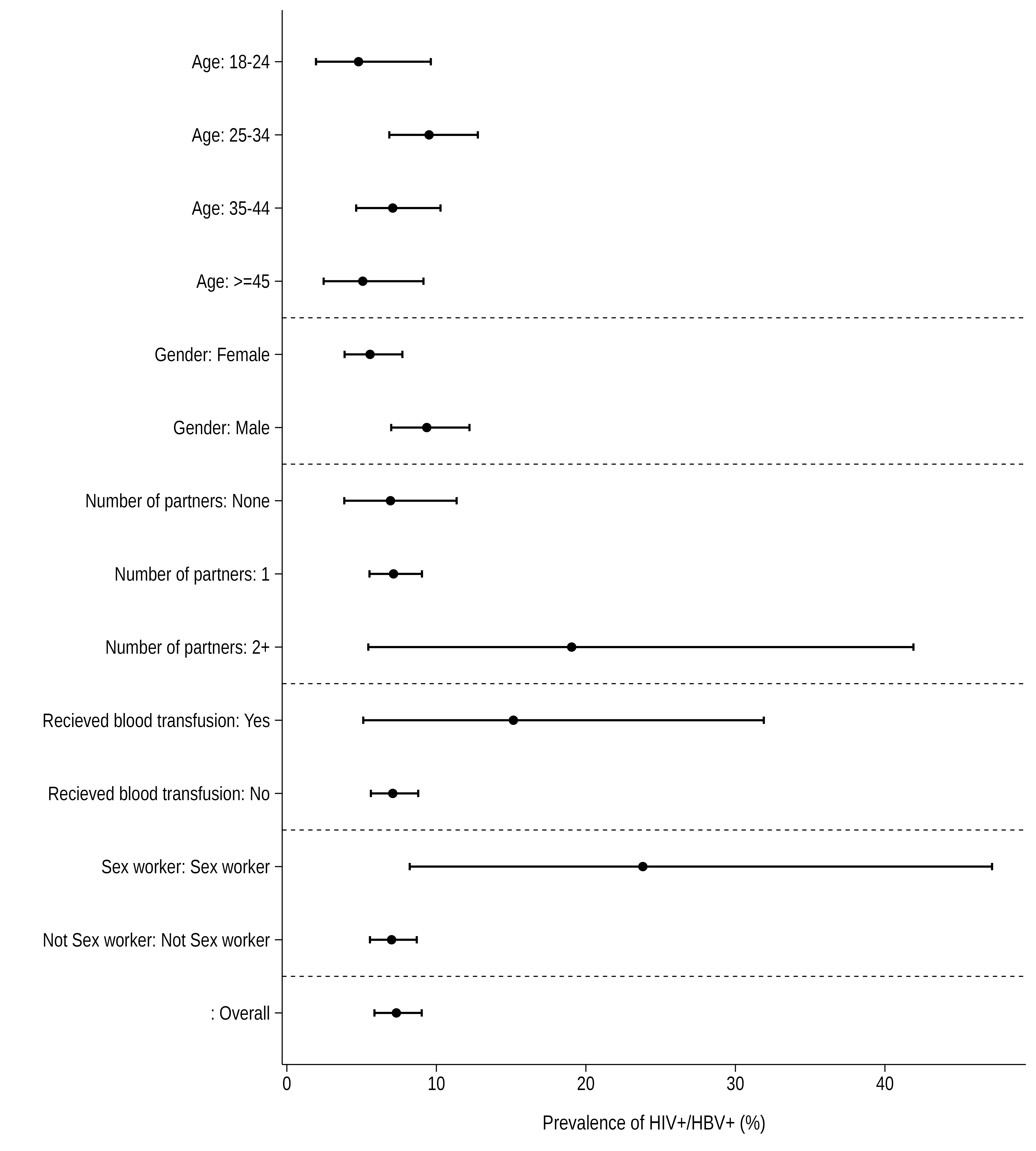


S1 Figure – prevalence of hepatitis B among patients with HIV per selected characteristics.

S1 Table - Socio-demographic characteristics per gender

| **Characteristic** | **Overall**  N = 1106 (100.0%) | **Male**  N = 513 (100.0%) | **Female**  N = 593 (100.0%) |
| --- | --- | --- | --- |
| **Age (years)** |  |  |  |
| Median (IQR) | 34 (28, 42) | 37 (31, 42) | 32 (26, 40) |
| **Born after 2001, n (%)** |  |  |  |
| No | 1,048 (94.76%) | 496 (96.69%) | 552 (93.09%) |
| Yes | 58 (5.24%) | 17 (3.31%) | 41 (6.91%) |
| **Level of education, n (%)** |  |  |  |
| Primary / Illiteracy | 526 (47.56%) | 243 (47.37%) | 283 (47.72%) |
| Secondary / Higher | 580 (52.44%) | 270 (52.63%) | 310 (52.28%) |
| **Marital Status, n (%)** |  |  |  |
| Single | 480 (43.40%) | 206 (40.16%) | 274 (46.21%) |
| Married | 594 (53.71%) | 300 (58.48%) | 294 (49.58%) |
| Divorced | 32 (2.89%) | 7 (1.36%) | 25 (4.22%) |
| **Unprotected sexual intercourse in the last 6 months, n (%)** |  |  |  |
| Yes | 525 (47.47%) | 258 (50.29%) | 267 (45.03%) |
| No | 581 (52.53%) | 255 (49.71%) | 326 (54.97%) |
| **Sex worker, n (%)** |  |  |  |
| Yes | 21 (1.90%) | 7 (1.36%) | 14 (2.36%) |
| No | 1,085 (98.10%) | 506 (98.64%) | 579 (97.64%) |
| **Scarification, n (%)** |  |  |  |
| Yes | 38 (3.44%) | 17 (3.31%) | 21 (3.54%) |
| No | 1,068 (96.56%) | 496 (96.69%) | 572 (96.46%) |
| **Tattoo/piercings, n (%)** |  |  |  |
| Yes | 193 (17.45%) | 104 (20.27%) | 89 (15.01%) |
| No | 913 (82.55%) | 409 (79.73%) | 504 (84.99%) |
| **Received blood transfusion, n (%)** |  |  |  |
| Yes | 33 (2.98%) | 12 (2.34%) | 21 (3.54%) |
| No | 1,073 (97.02%) | 501 (97.66%) | 572 (96.46%) |
| **Prior HBV vaccination, n (%)** |  |  |  |
| Yes | 56 (5.06%) | 19 (3.70%) | 37 (6.24%) |
| No | 1,050 (94.94%) | 494 (96.30%) | 556 (93.76%) |
| **Alcohol consumption, n (%)** |  |  |  |
| Yes | 556 (50.27%) | 315 (61.40%) | 241 (40.64%) |
| No | 550 (49.73%) | 198 (38.60%) | 352 (59.36%) |
| **Number of partners, n (%)** |  |  |  |
| None | 202 (18.26%) | 84 (16.37%) | 118 (19.90%) |
| 1 | 883 (79.84%) | 414 (80.70%) | 469 (79.09%) |
| >=2 | 21 (1.90%) | 15 (2.92%) | 6 (1.01%) |

S2 Table - Socio-demographic characteristics per gender among HIV+/HBV+

| **Characteristic** | **Overall**  N = 81 (100.0%) | **Male**  N = 48 (100.0%) | **Female**  N = 33 (100.0%) |
| --- | --- | --- | --- |
| **Age (years)** |  |  |  |
| Median (IQR) | 34 (30, 39) | 36 (32, 42) | 32 (26, 36) |
| **Born after 2001, n (%)** |  |  |  |
| No | 78 (96.30%) | 48 (100.00%) | 30 (90.91%) |
| Yes | 3 (3.70%) | 0 (0.00%) | 3 (9.09%) |
| **Level of education, n (%)** |  |  |  |
| Primary / Illiteracy | 39 (48.15%) | 27 (56.25%) | 12 (36.36%) |
| Secondary / Higher | 42 (51.85%) | 21 (43.75%) | 21 (63.64%) |
| **Marital Status, n (%)** |  |  |  |
| Single | 33 (40.74%) | 20 (41.67%) | 13 (39.39%) |
| Married | 46 (56.79%) | 28 (58.33%) | 18 (54.55%) |
| Divorced | 2 (2.47%) | 0 (0.00%) | 2 (6.06%) |
| **Unprotected sexual intercourse in the last 6 months, n (%)** |  |  |  |
| Yes | 40 (49.38%) | 24 (50.00%) | 16 (48.48%) |
| No | 41 (50.62%) | 24 (50.00%) | 17 (51.52%) |
| **Sex worker, n (%)** |  |  |  |
| Yes | 5 (6.17%) | 2 (4.17%) | 3 (9.09%) |
| No | 76 (93.83%) | 46 (95.83%) | 30 (90.91%) |
| **Scarification, n (%)** |  |  |  |
| Yes | 4 (4.94%) | 2 (4.17%) | 2 (6.06%) |
| No | 77 (95.06%) | 46 (95.83%) | 31 (93.94%) |
| **Tattoo/piercings, n (%)** |  |  |  |
| Yes | 19 (23.46%) | 10 (20.83%) | 9 (27.27%) |
| No | 62 (76.54%) | 38 (79.17%) | 24 (72.73%) |
| **Received blood transfusion, n (%)** |  |  |  |
| Yes | 5 (6.17%) | 3 (6.25%) | 2 (6.06%) |
| No | 76 (93.83%) | 45 (93.75%) | 31 (93.94%) |
| **Prior HBV vaccination, n (%)** |  |  |  |
| Yes | 3 (3.70%) | 0 (0.00%) | 3 (9.09%) |
| No | 78 (96.30%) | 48 (100.00%) | 30 (90.91%) |
| **Alcohol consumption, n (%)** |  |  |  |
| Yes | 47 (58.02%) | 32 (66.67%) | 15 (45.45%) |
| No | 34 (41.98%) | 16 (33.33%) | 18 (54.55%) |
| **Number of partners, n (%)** |  |  |  |
| None | 14 (17.28%) | 9 (18.75%) | 5 (15.15%) |
| 1 | 63 (77.78%) | 38 (79.17%) | 25 (75.76%) |
| >=2 | 4 (4.94%) | 1 (2.08%) | 3 (9.09%) |

S3 Table – Socio-demographic characteristics of individuals aged between 18 and 24 years by whether born before 2001 or after 2001

| **Characteristic** | **Overall**  N = 146 (100.0%) | **Born before 2001**  N = 88 (100.0%) | **Born on or after 2001**  N = 58 (100.0%) |
| --- | --- | --- | --- |
| **Age (years)** |  |  |  |
| Median (IQR) | 22 (20, 23) | 23 (22, 24) | 20 (19, 20) |
| **Gender, n (%)** |  |  |  |
| Male | 39 (26.71%) | 22 (25.00%) | 17 (29.31%) |
| Female | 107 (73.29%) | 66 (75.00%) | 41 (70.69%) |
| **Level of education, n (%)** |  |  |  |
| Primary / Illiteracy | 40 (27.40%) | 23 (26.14%) | 17 (29.31%) |
| Secondary / Higher | 106 (72.60%) | 65 (73.86%) | 41 (70.69%) |
| **Marital Status, n (%)** |  |  |  |
| Single | 96 (65.75%) | 54 (61.36%) | 42 (72.41%) |
| Married | 50 (34.25%) | 34 (38.64%) | 16 (27.59%) |
| Divorced | 0 (0.00%) | 0 (0.00%) | 0 (0.00%) |
| **Unprotected sexual intercourse in the last 6 months, n (%)** |  |  |  |
| Yes | 64 (43.84%) | 39 (44.32%) | 25 (43.10%) |
| No | 82 (56.16%) | 49 (55.68%) | 33 (56.90%) |
| **Sex worker, n (%)** |  |  |  |
| Yes | 4 (2.74%) | 4 (4.55%) | 0 (0.00%) |
| No | 142 (97.26%) | 84 (95.45%) | 58 (100.00%) |
| **Scarification, n (%)** |  |  |  |
| Yes | 4 (2.74%) | 2 (2.27%) | 2 (3.45%) |
| No | 142 (97.26%) | 86 (97.73%) | 56 (96.55%) |
| **Tattoo/piercings, n (%)** |  |  |  |
| Yes | 19 (13.01%) | 12 (13.64%) | 7 (12.07%) |
| No | 127 (86.99%) | 76 (86.36%) | 51 (87.93%) |
| **Received blood transfusion, n (%)** |  |  |  |
| Yes | 7 (4.79%) | 6 (6.82%) | 1 (1.72%) |
| No | 139 (95.21%) | 82 (93.18%) | 57 (98.28%) |
| **Prior HBV vaccination, n (%)** |  |  |  |
| Yes | 51 (34.93%) | 0 (0.00%) | 51 (87.93%) |
| No | 95 (65.07%) | 88 (100.00%) | 7 (12.07%) |
| **Alcohol consumption, n (%)** |  |  |  |
| Yes | 59 (40.41%) | 41 (46.59%) | 18 (31.03%) |
| No | 87 (59.59%) | 47 (53.41%) | 40 (68.97%) |
| **Number of partners, n (%)** |  |  |  |
| None | 29 (19.86%) | 17 (19.32%) | 12 (20.69%) |
| 1 | 115 (78.77%) | 69 (78.41%) | 46 (79.31%) |
| >=2 | 2 (1.37%) | 2 (2.27%) | 0 (0.00%) |
| **ahbs, n (%)** |  |  |  |
| HIV+/HBV- | 139 (95.21%) | 84 (95.45%) | 55 (94.83%) |
| HIV+/HBV+ | 7 (4.79%) | 4 (4.55%) | 3 (5.17%) |

S4 Table – Prevalence of hepatitis B among people with HIV between 18 and 24 years at recruitment

|  | | **HIV and HIV/HBV co-infection** | |  |  |
| --- | --- | --- | --- | --- | --- |
| **Characteristic** | **Overall** | **HIV+/HBV-** | **HIV+/HBV+** | **Prevalence of Coinfection (%)^*^** | **95% Confidence Interval** |
|  | N (%) | N (%) | N (%) |  |  |
| **Total** | 146 (100.0) | 139 (100.0) | 7 (100.0) | 4.7 | 1.9 – 9.4 |
|  |  |  |  |  |  |
| 18-24 born before 2001 | 88 (60.3) | 84 (60.4) | 4 (57.1) | 4.5 | 1.3 – 11.2 |
| 18-24 born on or after 2001 | 58 (39.7) | 55 (39.6) | 3 (42.9) | 5.1 | 1.1 – 14.4 |
|  |  |  |  |  |  |

* Prevalence of coinfection is the proportion of HBsAg positive among HIV patients.

S5 Table – Association between factors and the prevalence of HIV+/HBV+ coinfection using prevalence ratios (PR)

|  | **Unadjusted^*^** | | | | | **Adjusted^*^** | | | | |
| --- | --- | --- | --- | --- | --- | --- | --- | --- | --- | --- |
| **Predictor** |  | **PR**^1^ | **95% CI**^1^ | **p-value** |  | **PR**^1^ | **95% CI**^1^ | **p-value** |  | |
| **Age (years)** |  |  |  | 0.134 |  |  |  | 0.205 |  | |
| 18-24 |  | 0.50 | 0.21, 1.03 | 0.086 |  | 0.56 | 0.23, 1.37 | 0.203 |  | |
| 25-34 |  | 1.00 | — |  |  | — | — |  |  | |
| 35-44 |  | 0.74 | 0.45, 1.20 | 0.230 |  | 0.72 | 0.44, 1.17 | 0.188 |  | |
| >=45 |  | 0.53 | 0.26, 1.00 | 0.068 |  | 0.53 | 0.26, 1.10 | 0.087 |  | |
| **Gender** |  |  |  |  |  |  |  |  |  | |
| Female |  | 1.00 | — |  |  | — | — |  |  | |
| Male |  | 1.68 | 1.10, 2.60 | **0.017** |  | 1.63 | 1.04, 2.55 | **0.031** |  | |
| **Level of education** |  |  |  |  |  |  |  |  |  | |
| Primary / Illiteracy |  | 1.00 | — |  |  | — | — |  |  | |
| Secondary / Higher |  | 0.98 | 0.64, 1.49 | 0.912 |  | 0.94 | 0.62, 1.44 | 0.784 |  | |
| **Marital Status** |  |  |  | 0.839 |  |  |  | 0.857 |  | |
| Single |  | 1.00 | — |  |  | — | — |  |  | |
| Married |  | 1.13 | 0.74, 1.75 | 0.588 |  | 1.11 | 0.71, 1.74 | 0.642 |  | |
| Divorced |  | 0.91 | 0.15, 2.80 | 0.893 |  | 1.33 | 0.31, 5.65 | 0.700 |  | |
| **Number of partners** |  |  |  | 0.103 |  |  |  | 0.277 |  | |
| None |  | 1.00 | — |  |  | — | — |  |  | |
| 1 |  | 1.03 | 0.61, 1.88 | 0.919 |  | 0.90 | 0.50, 1.63 | 0.735 |  | |
| >=2 |  | 2.75 | 0.84, 6.90 | 0.051 |  | 1.97 | 0.65, 5.91 | 0.228 |  | |
| **HIV partner status** |  |  |  | 0.484 |  |  |  |  |  | |
| Positive |  | 1.69 | 0.75, 4.51 | 0.242 |  |  |  |  |  | |
| Negative |  | 1.00 | — |  |  |  |  |  |  | |
| Unknown |  | 1.41 | 0.66, 3.64 | 0.418 |  |  |  |  |  | |
| **Unprotected sexual intercourse in the last 6 months** |  |  |  |  |  |  |  |  |  | |
| No |  | 1.00 | — |  |  | — | — |  |  | |
| Yes |  | 1.08 | 0.71, 1.64 | 0.720 |  | 0.92 | 0.60, 1.43 | 0.726 |  | |
| **Sex worker** |  |  |  |  |  |  |  |  |  | |
| No |  | 1.00 | — |  |  | — | — |  |  | |
| Yes |  | 3.40 | 1.30, 6.63 | **0.003** |  | 3.04 | 1.37, 6.72 | **0.006** |  | |
| **Scarification** |  |  |  |  |  |  |  |  |  | |
| No |  | 1.00 | — |  |  | — | — |  |  | |
| Yes |  | 1.46 | 0.46, 3.28 | 0.436 |  | 0.87 | 0.31, 2.48 | 0.798 |  | |
| **Tattoo/piercings** |  |  |  |  |  |  |  |  |  | |
| No |  | 1.00 | — |  |  | — | — |  |  | |
| Yes |  | 1.45 | 0.86, 2.31 | 0.137 |  | 1.20 | 0.72, 2.02 | 0.487 |  | |
| **Blood transfusion** |  |  |  |  |  |  |  |  |  | |
| No |  | 1.00 | — |  |  | — | — |  |  | |
| Yes |  | 2.14 | 0.79, 4.38 | 0.074 |  | 2.04 | 0.92, 4.54 | 0.080 |  | |
| **Prior HBV vaccination** |  |  |  |  |  |  |  |  |  | |
| No |  | 1.00 | — |  |  | — | — |  |  | |
| Yes |  | 0.72 | 0.18, 1.85 | 0.564 |  | 1.09 | 0.30, 4.01 | 0.894 |  | |
| **Alcohol consumption** |  |  |  |  |  |  |  |  |  | |
| No |  | 1.00 | — |  |  | — | — |  |  | |
| Yes |  | 1.37 | 0.90, 2.11 | 0.149 |  | 1.14 | 0.73, 1.79 | 0.573 |  | |
| ^1^PR – Prevalence Ratio, 95% CI – 95% Confidence Interval  * The unadjusted and adjusted PR were estimated through log-binomial regression models. | | | | | | | | | |  |
|  | | | | | | | | | |  |

S6 Table - Clinical characteristics of study subjects and hepatitis B prevalence

|  | | **HIV and HIV/HBV co-infection** | |  |  |
| --- | --- | --- | --- | --- | --- |
| **Characteristic** | **Overall** | **HIV+/HBV-** | **HIV+/HBV+** | **Proportion of Coinfected (%)** | **95% Confidence Interval** |
|  | N (%) | N (%) | N (%) |  |  |
| **Total** | 1,106 (100.0) | 1,025 (100.0) | 81 (100.0) | 7.3 | 5.9 – 9.0 |
| **BMI (Kg/m2), Mean (SD)** | 23.6 (4.4) | 23.7 (4.5) | 22.9 (3.9) |  |  |
| **BMI (Kg/m2), n (%)** |  |  |  |  |  |
| < 18 | 61 (5.5) | 56 (5.5) | 5 (6.2) | 8.2 | 2.7 – 18.1 |
| 18-25 | 721 (65.2) | 665 (64.9) | 56 (69.1) | 7.8 | 5.9 – 10.0 |
| 25-30 | 214 (19.4) | 199 (19.4) | 15 (18.5) | 7.0 | 4.0 – 11.3 |
| > 30 | 110 (10.0) | 105 (10.2) | 5 (6.2) | 4.6 | 1.5 – 10.3 |
| **Jaundice, n (%)** |  |  |  |  |  |
| Yes | 10 (0.9) | 6 (0.6) | 4 (4.9) | 40.0 | 12.2 – 73.8 |
| No | 1,096 (99.1) | 1,019 (99.4) | 77 (95.1) | 7.0 | 5.6 – 8.7 |
| **Ascites, n (%)** |  |  |  |  |  |
| Yes | 4 (0.4) | 1 (0.1) | 3 (3.70) | 75.0 | 19.4 – 99.4 |
| No | 1,102 (99.6) | 1,024 (99.9) | 78 (96.3) | 7.1 | 5.6 – 8.8 |
| **Hepatomegaly, n (%)** |  |  |  |  |  |
| Yes | 11 (0.99) | 3 (0.29) | 8 (9.9) | 72.7 | 39.0 – 94.0 |
| No | 1,095 (99.01) | 1,022 (99.71) | 73 (90.1) | 6.7 | 5.3 – 8.3 |
| **Splenomegaly, n (%)** |  |  |  |  |  |
| No | 1,106 (100.0) | 1,025 (100.0) | 81 (100.0) | 7.3 | 5.9 – 9.0 |

S7 Table - Laboratory characteristics of study subjects

| **Characteristic** | **Overall** | **HIV+/HBV-** | **HIV+/HBV+** | **p-value** |
| --- | --- | --- | --- | --- |
|  | N (%) | N (%) | N (%) |  |
| **Total** | 1,106 (100)^1^ | 1,025 (92.7) | 81 (7.3) |  |
| **Platelets count** Median (IQR) | 224.5 (171.0, 285.0) | 226.0 (173.0, 286.0) | 216.0 (134.0, 274.0) | 0.146^2^ |
| **CD4+T – cell count, cells/mm3** Median (IQR) | 251.0 (137.3, 419.8) | 252.0 (140.0, 421.0) | 238.0 (129.0, 367.0) | 0.486^2^ |
| **CD4+T** |  |  |  | 0.615^3^ |
| > 500 | 192 (17.4%) | 180 (17.6%) | 12 (14.8%) |  |
| 200 – 500 | 482 (43.6%) | 446 (43.5%) | 36 (44.4%) |  |
| 100 – 200 | 238 (21.5%) | 223 (21.8%) | 15 (18.5%) |  |
| < 100 | 194 (17.5%) | 176 (17.2%) | 18 (22.2%) |  |
| **AST, IU** Median (IQR) | 30.0 (23.4, 40.0) | 29.5 (23.0, 39.0) | 38.0 (27.9, 90.0) | <0.001^2^ |
| (Missing) | 5 | 5 | 0 |  |
| **AST, IU** |  |  |  | <0.001^3^ |
| <= 30 | 557 (50.6%) | 532 (52.2%) | 25 (30.9%) |  |
| > 30 | 544 (49.4%) | 488 (47.8%) | 56 (69.1%) |  |
| (Missing) | 5 | 5 | 0 |  |
| **ALT, IU** Median (IQR) | 21.8 (14.6, 35.2) | 21.5 (14.4, 33.6) | 32.4 (19.6, 59.8) | <0.001^2^ |
| (Missing) | 5 | 5 | 0 |  |
| **ALT, IU** |  |  |  | <0.001^3^ |
| <= 30 | 737 (66.9%) | 699 (68.5%) | 38 (46.9%) |  |
| > 30 | 364 (33.1%) | 321 (31.5%) | 43 (53.1%) |  |
| (Missing) | 5 | 5 | 0 |  |
| **GGT, IU** Median (IQR) | 31.4 (21.0, 51.0) | 30.2 (20.6, 48.3) | 45.6 (30.9, 78.0) | <0.001^2^ |
| (Missing) | 5 | 5 | 0 |  |
| **GGT, IU** |  |  |  | <0.001^3^ |
| <= 49 | 813 (73.8%) | 767 (75.2%) | 46 (56.8%) |  |
| > 49 | 288 (26.2%) | 253 (24.8%) | 35 (43.2%) |  |
| (Missing) | 5 | 5 | 0 |  |
| **ALP, IU** Median (IQR) | 61.4 (46.5, 82.0) | 61.0 (46.0, 80.4) | 75.0 (51.8, 99.0) | 0.002^2^ |
| (Missing) | 98 | 90 | 8 |  |
| **ALP, IU** |  |  |  | <0.001^4^ |
| <= 120 | 943 (93.6%) | 883 (94.4%) | 60 (82.2%) |  |
| > 120 | 65 (6.4%) | 52 (5.6%) | 13 (17.8%) |  |
| (Missing) | 98 | 90 | 8 |  |
| **eGFR estimated glomerular filtration rate (mL/min/1.73m2)** Median (IQR) | 118.1 (96.4, 130.9) | 118.1 (96.4, 130.9) | 116.9 (98.4, 130.6) | 0.820^2^ |
| **eGFR estimated glomerular filtration rate (mL/min/1.73m2)** |  |  |  | 0.814^4^ |
| < 30 | 38 (3.5%) | 36 (3.5%) | 2 (2.5%) |  |
| 30-50 | 32 (2.9%) | 31 (3.0%) | 1 (1.2%) |  |
| > 50 | 1,031 (93.6%) | 953 (93.4%) | 78 (96.3%) |  |
| (Missing) | 5 | 5 | 0 |  |
| **HIV – RNA (cp/mL) (log10)** Median (IQR) | 4.7 (4.0, 5.2) | 4.7 (4.0, 5.2) | 4.8 (4.0, 5.1) | 0.982^2^ |
| (Missing) | 24 | 24 | 0 |  |
| **HIV – RNA (cp/mL)** |  |  |  | 0.595^4^ |
| Undetectable | 47 (4.3%) | 43 (4.3%) | 4 (4.9%) |  |
| < 300 | 63 (5.8%) | 57 (5.7%) | 6 (7.4%) |  |
| 300 – 1000 | 40 (3.7%) | 39 (3.9%) | 1 (1.2%) |  |
| > 1000 | 932 (86.1%) | 862 (86.1%) | 70 (86.4%) |  |
| (Missing) | 24 | 24 | 0 |  |
| **APRI** Median (IQR) | 0.4 (0.2, 0.5) | 0.3 (0.2, 0.5) | 0.5 (0.3, 1.1) | <0.001^2^ |
| (Missing) | 5 | 5 | 0 |  |
| **APRI** |  |  |  | <0.001^3^ |
| <=0.5 | 791 (71.8%) | 750 (73.5%) | 41 (50.6%) |  |
| 0.5 – 1.0 | 310 (28.2%) | 270 (26.5%) | 40 (49.4%) |  |
| >1.0 |  |  |  |  |
| (Missing) | 5 | 5 | 0 |  |
| **FIB-4** Median (IQR) | 1.0 (0.6, 1.7) | 1.0 (0.6, 1.6) | 1.3 (0.8, 2.3) | 0.004^2^ |
| (Missing) | 5 | 5 | 0 |  |
| **FIB-4** |  |  |  | 0.013^3^ |
| <=3.25 | 1,016 (92.3%) | 947 (92.8%) | 69 (85.2%) |  |
| >3.25 | 85 (7.7%) | 73 (7.2%) | 12 (14.8%) |  |
| (Missing) | 5 | 5 | 0 |  |
| ^1^n (%) | | | | |
| ^2^Wilcoxon rank sum test | | | | |
| ^3^Pearson's Chi-squared test | | | | |
| ^4^Fisher's exact test | | | | |
